# Some Common Diseases of the Eye: Their Diagnosis and Treatment

**Published:** 1908-07-11

**Authors:** Percy Dunn

**Affiliations:** Senior Ophthalmic Surgeon to the West London Hospital.


### Hospital Clinics.

### SOME COMMON DISEASES OF THE EYE.

### THEIR DIAGNOSIS AND TREATMENT.

By PERCY DUNN, F.R.C.S., Senior Ophthalmic Surgeon to the West London Hospital.

A Post-graduate Lecture Delivered at the West London Post-graduate College.

Gentlemen,—I want you to imagine that this is my out-patient room, and that you have come to see and examine with me some of the cases which present themselves for treatment. In order to facilitate this imaginary proceeding I have brought —and will pass round—a few coloured drawings showing some of the common diseases of the eye taken from Haab's Atlas, which will very readily serve as the counterfeit presentments of the patients whom we may imagine to be present.

#### Simple Conjunctivitis.

Let us suppose that the first case which comes for treatment is that of a young woman. There is some redness of the eyelids of both eyes; the lashes are adherent with dried mucus; at the inner canthus some white mucous secretion is plainly visible. On raising the lids there is to be seen marked infection of the conjunctiva. The patient then states that the lids are always "gummed together" in the morning; that she feels "as if there were sand in the eyes." The diagnosis is thus made plain, both by the appearances presented as well as by the statement of the patient; but for our purpose let us examine the case a little further. And here let it be borne in mind that the most prominent condition present—namely, injection of the globe—is merely a symptom, not a disease, and that it is coincident with many other diseases, such as iritis and ulcers of the cornea. To make, therefore, a mistake in the differential diagnosis would be attended with most untoward consequences to the patient.

In all cases of doubt close inquiry should be made into the cause of the injection, and with this object in view the eyes should be examined by the oblique or focal illumination, and especially so when only one eye happens to be the seat of inflammation. As showing the importance of this, quite recently I have seen in private two cases in which an inflamed eye was being wrongly treated for conjunctivitis: in one case I found a small foreign body upon the cornea, and in the other a foreign body beneath the upper lid. In simple conjunctival inflammation the injected vessels are seen to be superficial; they can be moved freely over the sclerotic, and slight pressure with the finger empties them of their blood; the redness is of a dull brick colour, and is more marked towards the periphery of the eyeball. Whereas, should the injection be mainly ciliary in origin, such as occurs in iritis and corneal ulcers, the redness is bright pink in colour, is mainly centred around the cornea, and the distension of the vessels is not affected either by movement or pressure of the finger. In the case before us, then, we have to deal with one of simple conjunctivitis.

#### Bacteriology and Treatment.

Bacteriology has shown that simple conjunctivitis is a disease in which no fewer than five special varieties of micro-organisms may be concerned. The most common variety is that known as the Koch-Weeks bacillus; but the disease may also be caused by the streptococcus, the staphylococcus, the pneumococcus of Fraenkel, and the Morax-Axenfeld diplobacillus. Despite, however, the complexity of origin, there is no difference in the clinical type of the disease.

Given, then, a disease of such pronounced bacillary origin as simple conjunctivitis, possibly it may be thought that the treatment would undoubtedly demand the use of some powerful germicide in order to destroy the infection. But bear in mind that the conjunctival sac is not in any sense a region which can indiscriminately be dealt with by powerful germicides, however much these may be indicated in the treatment of its particular diseases. To this matter I shall again refer later on, when discussing the question of the treatment of purulent conjunctivitis. Seeing, then, that the cause of simple conjunctival inflammation is incapable of being directly dealt with, germicides as such are not indicated in the treatment. On the other hand, astringents are invaluable. The effect of astringents is doubtless to cause contraction of the blood-vessels, and thus to change the conditions present in the conjunctival sac, and to destroy for the time being its capacity for serving as a culture medium for micro-organisms. In the imaginary case, then, of the woman before us, the treatment indicated is a lotion of one grain of sulphate of zinc to the ounce of water, to be used twice daily, together with a simple ointment to be applied to the lids at bedtime to prevent them from adhering.

#### Meibomian Cysts.

The next case that we will imagine is that of a young girl of twenty. As she comes forward for examination she points to the upper lid of the right eye, upon which is plainly to be seen a small swelling, which she asserts has been present for a month and is growing larger. In this coloured drawing is aptly delineated the condition which the lid presents. The swelling is known as a meibomian cyst. It is not inflamed nor painful, and the only serious feature about it is its unsightliness. These cystlike formations may occur at any age. Commonly they are met with in young girls who are anæmic and constipated. Sometimes their early development is associated with slight inflammatory signs, which, however, soon pass off. This simple affection is due to a chronic inflammation of the meibomian glands, in the course of which granulation tissue is formed, enclosed by a capsule of connective tissue. Later on the central portion of the granulation tissue undergoes mucoid degeneration, leaving the cavity thereof occupied by contents of a semi-liquid consistence. On the inner side of the everted lid, the conjunctiva, as displayed by this drawing, becomes injected, thickened, uneven, and somewhat bulging. It is owing to the unevenness and thickening of the conjunctiva that these meibomian formations sometimes give rise to signs of slight general conjunctival irritation, and the discomfort thus caused leads the patient at last to seek advice.

### Their Treatment.

The treatment of these cases is simple and effectual. A four-per-cent. solution of cocaine should be instilled into the conjunctival sac. Then the upper lid is everted and a Beer's knife made to transfix the swelling in a horizontal direction. In books the incision is generally directed to be made vertically; but for obvious reasons the horizontal direction is the best—a detail worthy of note. While mentioning the word detail let me remind you that this small procedure comes under the category of an operation: it is so regarded by the patient because a knife is used. Trivial though the procedure may be, nevertheless, as a matter of principle, the routine as to antiseptic precautions should be strictly followed. Therefore, after the instillation of cocaine, the lids and conjunctival sac should be thoroughly douched with a warm 1-4000 solution of chinosol: the swabs should consist of chinosol gauze, soaked in a solution of the same strength, and the instruments should be sterilised by immersing them for some moments in absolute phenol, and afterwards placed in a 1-20 solution of carbolic acid. The incision having been made, Davel's scoop is taken, thrust into the wound, and turned round several times so as to evacuate the contents and lacerate the environing capsule. Lastly, the wound is cleansed with the swabs and the conjunctival sac douched with the chinosol solution. The lid is replaced in position; no dressing is required. At first the swelling appears to be as large as before: that is due to the distension of the small cavity with blood, which, however, soon becomes absorbed. In order to facilitate this process it is well to direct the patient to begin massage of the lid with ung. hydrarg. dil. about forty-eight hours afterwards—that is to say, when the small wound has ceased to become tender to pressure.

### Purulent Conjunctivitis in Infants.

The next case we may imagine is that of a baby brought by its mother. The disease from which it is suffering is, perhaps, the most important of all those which may come under notice during an ordinary afternoon in the out-patient room. Look at this coloured drawing. Note the redness and the swelling of the eyelids; the exudate of thick, creamy-looking, yellowish pus flowing from between the lids over the cheek. Truly may this disease be described as important, for owing either to neglect or mismanagement, or both, 30 per cent. of the blindness in the blind asylums in the world is due to the sequels of its incidence. The case is one of purulent conjunctivitis, the origin of which, as you all know, depends upon a highly infective micro-organism—namely the gonococcus of Neisser. The destructive effects of the disease are illustrated in the rapidity with which the cornea becomes involved. The peril to which the cornea in these cases is exposed is due to the mechanical arrest of its nutrition from the intensity of the conjunctival inflammation, not to the invasion of the corneal tissue by the gonococci. Even a few hours in the delay of treatment may be fraught with the most perilous consequences to vision. And yet let it be remembered that, serious in its results and fatal to the eye as this disease may be, its treatment presents no real difficulty. Whether the result of the attack will be good or bad depends upon the condition of the cornea at the time that the child first comes under skilled treatment. If the cornea be intact, clear, and natural, it may be safely affirmed, without exception, that the case is one which will do well—one in which the peril of blindness will be averted. Unfortunately, however, all of us can recall deplorable cases in which, through the loss of valuable time, the disease has been permitted to exert its fullest destructive effects.

### Methods of Examination.

Let us now consider the various details required to be observed in the examination and treatment of these cases. First as to the examination. In acute cases, with much swelling of the lids, the child should always be examined under chloroform when seen for the first time. This for two reasons: partly to abolish the resistance of the child, partly to avoid the use of force in exposing the cornea. The risk associated with the application of force under these circumstances is that of rendering complete a threatened perforation, followed by prolapse of the iris, and even extrusion of the lens. As soon as the child is under the anæsthetic, a swab should be taken and the eyelids thoroughly cleansed with a 1 in 2000 solution of chinosol. Then the lids may be raised and the cornea examined. Sometimes, in order to do this effectually, a retractor is necessary; but always avoid using an instrument in these cases, if possible. If a retractor is used, be careful that it never touches the cornea.

### Treatment in Infants.

The examination having been concluded and the cornea found to be clear, what is the treatment? The remedies required are three in number: (1) Drops of nitrate of silver, one grain to the ounce of water. (2) A lotion of sulphate of zinc, one grain to the ounce of water. (3) A simple ointment, such as the ung. cetacei. The drops are in bad cases instilled into the conjunctival sac every hour, or, as the infant's mother is informed, every time that the clock strikes. The sound of the clock reminds the parent of the duty which she owes to her offspring, with the alternative of blindness resulting as the consequence of her neglect. Every ten minutes the lotion should be used to wash the discharge away from its lids, or as often as it is seen to collect, and the ointment must be smeared round the lids to keep them from adhering, as well as to protect the tender skin of the lids and face from being excoriated by the remedies employed.

In the course of forty-eight hours, provided that the instructions given above are fully carried out, a great improvement will be noticed in the condition of the infant's eyes. First the swelling of the lids will be less; the discharge much diminished in quantity as well as changed in character. It will be thinner, less purulent, less virulently infective. The frequency of the instillation of the nitrate of silver drops may then be reduced —that is to say the drops may be used every two or three hours during the day only. Later, when the discharge reaches the muco-purulent stage the drops may be used three times daily; and as soon as the purulent character of the discharge ceases, which is generally in the course of ten days, they may be discontinued. Nevertheless, the daily use of the zinc lotion is indicated for some days longer.

### Purulent Conjunctivitis in Adults.

Time does not permit me to discuss the special treatment of those cases in which the cornea has become ulcerated as the result of purulent conjunctivitis in the infant, more especially as I desire to direct your attention to one or two points in the treatment of purulent conjunctivitis as met with in the adult—a disease which is provocative of much anxiety to the surgeon. There is first the question of the age of the patient. It has been alleged that among all the cases of purulent conjunctivitis treated at the Royal London Ophthalmic Hospital in patients over forty years of age no eye was saved. Doubtless in some degree the explanation of this depends upon the fact that the cornea is less well nourished in the adult as compared with the child. But we must also bear in mind that the conditions favourable for the full development of the disease in the former are much greater than is the case in the latter.

Thus can be explained the difficulty which always environs the treatment of purulent conjunctivitis in the adult. In the conjunctival sac of the adult there is a sub-epithelial layer of adenoid tissue, for which the gonococci exhibit a special predilection. In this tissue, for the most part, they locate themselves, and there they are more or less protected from the germicidal effects of remedies.

### Management of Adult Patients.

There are many points of practical interest and importance in the management of this dread disease in the adult. Time, however, does not permit me to deal with the subject at any length. Nevertheless, I may allude to a few points which I regard as of much importance. In the first place, bear in mind that while in the child the danger to the cornea arises from delay and neglect in applying the proper treatment, in the adult the cornea is imperilled by, on the one hand, the extreme chemosis, arresting its nutrition, and, on the other, the impossibility of controlling the progress of the disease.

The primary point of treatment, therefore, in the adult is to remove as far as possible all mechanical pressure upon the cornea, such as ensues from the swelling of the lids and the chemosis. This may be effected by dividing freely the outer canthus with scissors, and by radial snips with the scissors in the œdematous conjunctiva. There is generally free hæmorrhage after these procedures, and it should be encouraged, since it assists in reducing the blood-pressure in the eye and orbit. Furthermore, every hour some drops of a one grain per fluid ounce solution of nitrate of silver must be instilled into the conjunctival sac, together with some one-in-1000 solution of chloride of adrenalin. Twice daily, also, eserine drops should be used, of the strength of half a grain to the ounce of water; and, lastly, the chinosol fomentations should be applied to the affected eye every two hours. The usual advice given is to apply ice compresses in cases of purulent conjunctivitis in the adult. This treatment, however, I deem to be harmful. Cold applications are not indicated in any form of ocular disease, and they should be especially excluded from every variety of disease in which the cornea is involved. A full diet is always necessary in these cases, even to the extent of some alcoholic stimulant; also quinine and iron or iron and strychnine are helpful, to counteract the depressed nutrition, which is often present.

### Three Illustrative Cases in Adults.

Some of you, doubtless, have seen the young nurse who for the past few weeks has been attending my out-patient room. She was sent to me by the medical superintendent of one of the neighbouring Poor-law infirmaries. At the time of her first visit there did not seem to be the least prospect of saving the eye: partly owing to the sloughing of the upper part of the cornea, partly to the chemosis, which had practically reached the stage of solid œdema. The treatment which I have just delineated was faithfully carried out by the sister in charge of the case, under the supervision of the medical superintendent, and for a time there was some improvement. Nevertheless, the chemosis never became visibly less, despite scarification and division of the outer canthus, together with the other adjuncts of local treatment.

Then at times much complaint of acute onsets of pain were made, until at length it seemed that relief could only be obtained by enucleation. Quite suddenly, however, the chemosis began to subside, the conjunctival injection to diminish, and the ulceration of the cornea—which had proceeded to perforation—ceased and showed some evidences of repair. There is now a dense, flattened leucoma of the upper part of the cornea, and some haziness in the pupillary area. Nevertheless, the eye has been saved, with the retention of some vision. The eye became infected while the nurse was attending to an infant suffering from purulent conjunctivitis.

A few years ago I admitted into the hospital a middle-aged midwife whose right eye had become similarly infected. This was a remarkable case, inasmuch as, although the eye did well, despite some ulceration of the cornea, the woman ultimately died of pyæmic symptoms, after having had her left knee-joint opened, in which there was pus. As is well known, arthritic inflammation is occasionally met with in infants in whom purulent conjunctivitis is present.

Another interesting case was that of a young man whom I admitted for a mild attack of the disease. Within forty-eight hours he suddenly began to vomit; his temperature rose to 105° F., and it was very doubtful to what his symptoms could be attributed. In due course, however, he was found to be suffering from an acute attack of variola, which necessitated his immediate removal from the hospital. I scarcely expected to see the patient again—still less his affected eye. But one day he returned to show himself, and I had the satisfaction of learning that his eye had made a good recovery, despite the variolous attack.

